# Complete Genome Sequence of the Olive-Infecting Strain *Xylella fastidiosa* subsp. *pauca* De Donno

**DOI:** 10.1128/genomeA.00569-17

**Published:** 2017-07-06

**Authors:** Annalisa Giampetruzzi, Maria Saponari, Rodrigo P. P. Almeida, Salwa Essakhi, Donato Boscia, Giuliana Loconsole, Pasquale Saldarelli

**Affiliations:** a Department of Soil, Plant and Food Sciences, University of Bari Aldo Moro, Bari, Italy; b Institute for Sustainable Plant Protection, National Research Council (CNR), Bari, Italy; c Department of Environmental Science, Policy and Management, University of California, Berkeley, Berkeley, California, USA

## Abstract

We report here the complete and annotated genome sequence of the plant-pathogenic bacterium *Xylella fastidiosa* subsp. *pauca* strain De Donno. This strain was recovered from an olive tree severely affected by olive quick decline syndrome (OQDS), a devastating olive disease associated with *X. fastidiosa* infections in susceptible olive cultivars.

## GENOME ANNOUNCEMENT

In 2013, *Xylella fastidiosa* was detected in olive trees (*Olea europaea* L.) in southern Italy (Apulia region). It represented the first outbreak of this quarantine pathogen under field conditions in the European Union, and it was the first documented event of widespread infections in this plant species. Infected trees exhibit a severe disease termed olive quick decline syndrome (OQDS). Symptoms of OQDS include yellow and brown lesions on leaf tips and margins, extensive branch and twig dieback, and subsequent tree mortality (1). Genome data and multilocus sequence typing (MLST) analyses (2, 3) showed that olive-infecting isolates of *X. fastidiosa* were genetically related to subspecies *pauca* and all harbored the sequence type 53 (ST53). In this report, we describe the complete and finished genome sequence of *X. fastidiosa* subsp. *pauca* strain De Donno, selected among the ST53-cultured isolates recovered from OQDS-affected olive trees. This strain was cultured in June 2014 from a symptomatic olive tree (40.011389 N 18.048056 E); when mechanically inoculated in different olive cultivars under experimental conditions, it caused symptoms identical to those observed in contaminated olive groves (4).

A combined strategy of sequencing by the HiSeq 4000 Illumina platform and PacBio RSII platform was performed. Illumina sequencing yielded a total of 5,700,601 2 × 150-bp high-quality paired reads, of which 1% (87,950 reads) low-quality reads were discarded. In parallel, 105,585 fastq reads, with a mean length of 8,527 bp (longest read, 56,602 bp), were obtained by PacBio sequencing. *De novo* hybrid genome assembly was done using both Illumina and PacBio data set with SPAdes version 3.9.0 (5, 6). The final assembly resulted in a single circular 2,508,465-nucleotide (nt) chromosome with 52% G+C content. In addition, a circular plasmid of 35,273 nt, named pXF-De Donno, with a G+C content of 49.6%, was also identified. Nucleotide coverage was, on average, 1,765.5× for the plasmid (standard deviation [SD], 216.9×) and 636.5× for the chromosome (SD, 76.2×). Functional annotation by submission to the NCBI Prokaryotic Genome Automatic Annotation Pipeline (PGAAP) resulted in the identification of 6 rRNA genes (2 operons), 49 tRNA loci, 2,381 genes, 2,322 protein-coding genes, 3 noncoding RNAs in the chromosome, and 39 protein-coding genes in the plasmid. The complete genome description of the strain De Donno offers insights into the biology of this devastating olive disease.

### Accession number(s).

This whole-genome shotgun project has been deposited in GenBank under the accession numbers CP020870 to CP020871. The version described in this paper is the first version.
